# Supplementary figures and images for: Translational repression of viral RNAs supports persistent arbovirus infection in mosquitoes
Source: PLoS Biol. 2026 Apr 28;24(4):e3003702. doi: 10.1371/journal.pbio.3003702 (PMC13124052; doi:10.1371/journal.pbio.3003702)

Fig 1D

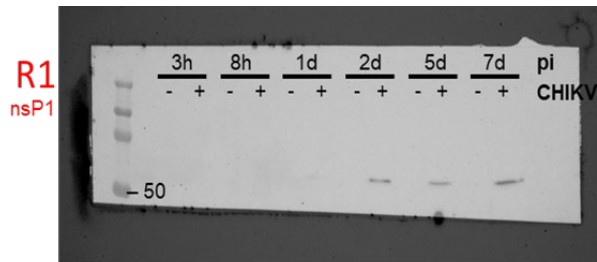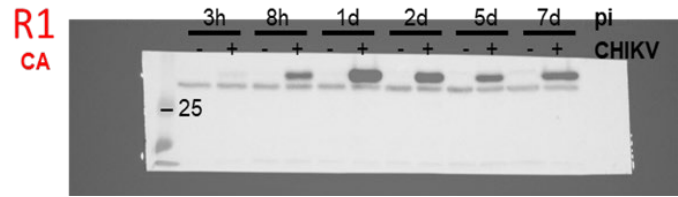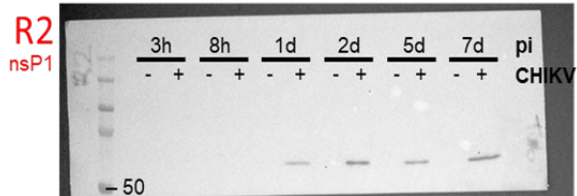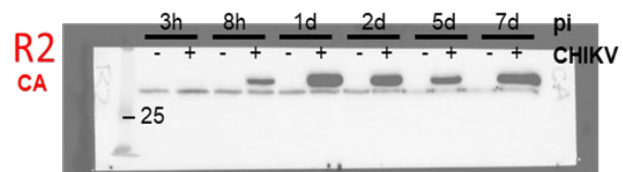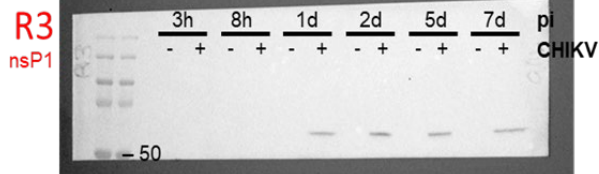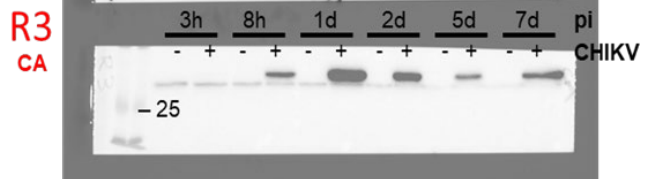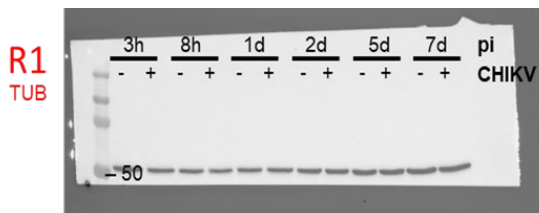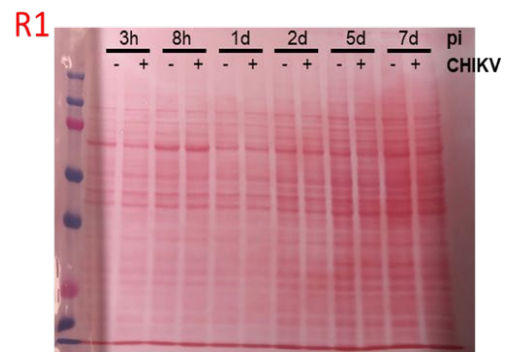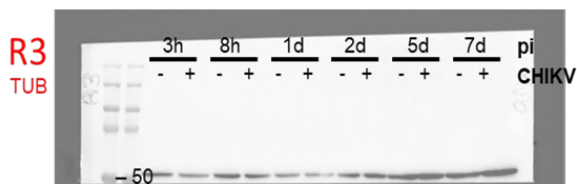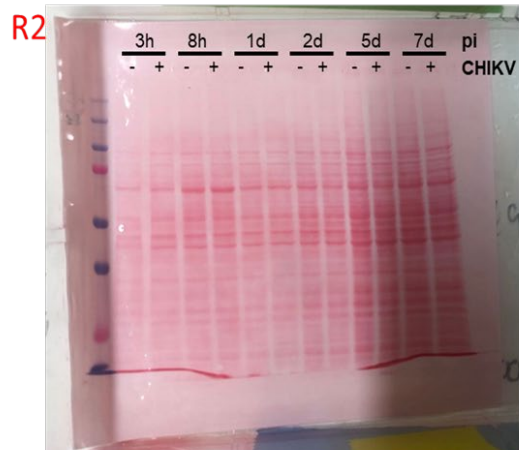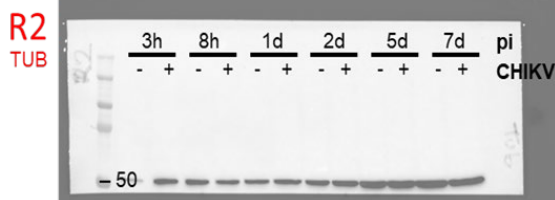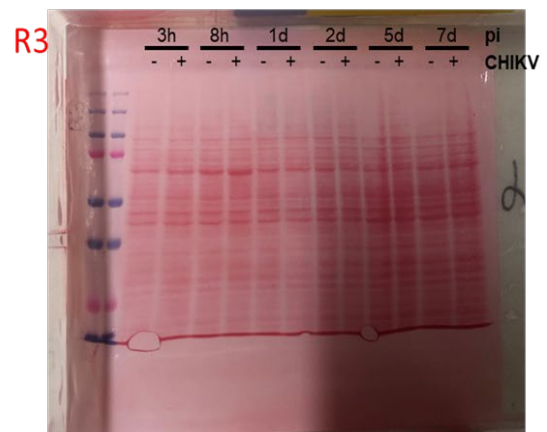

Fig 1E

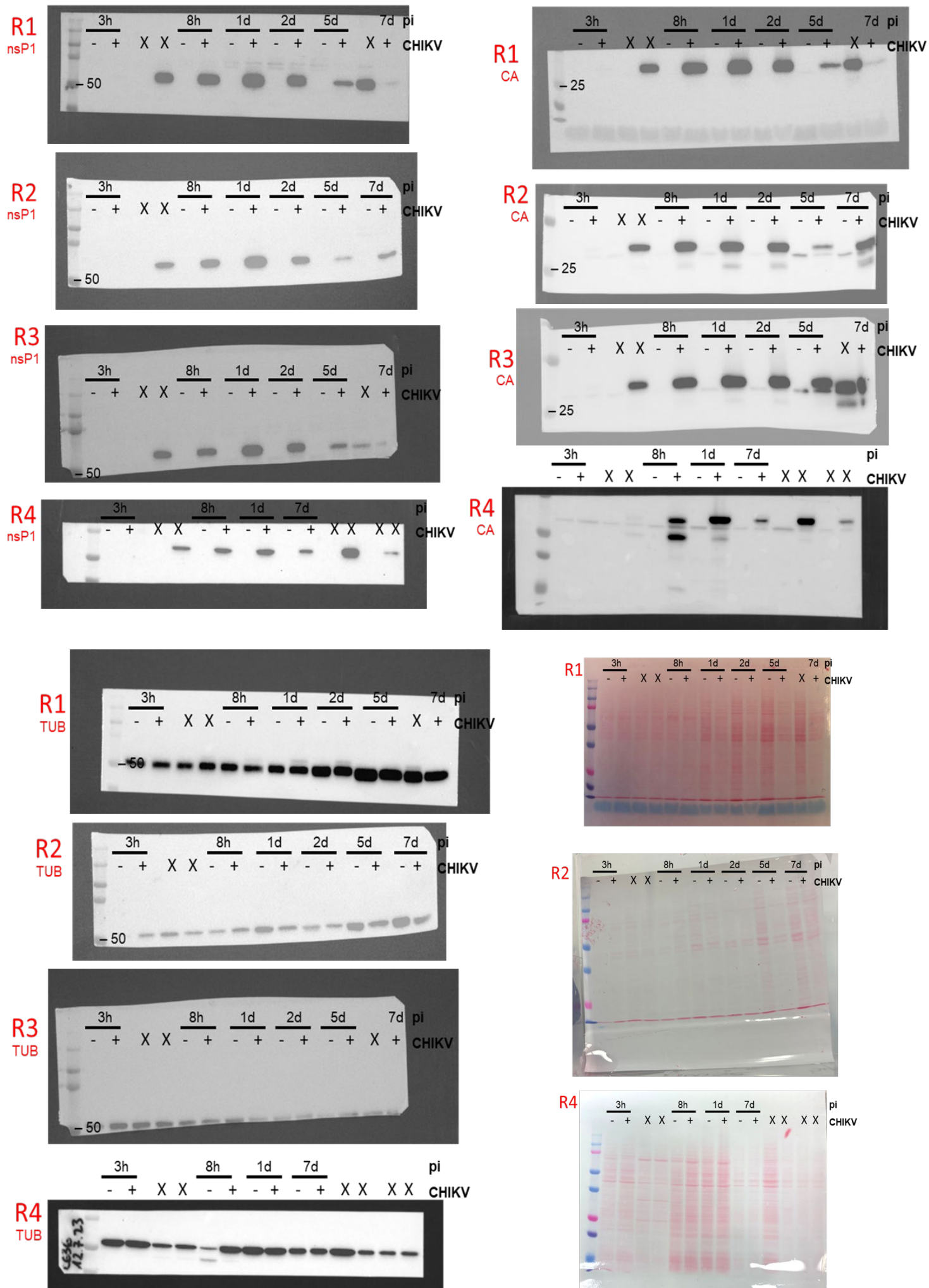

Fig 2E

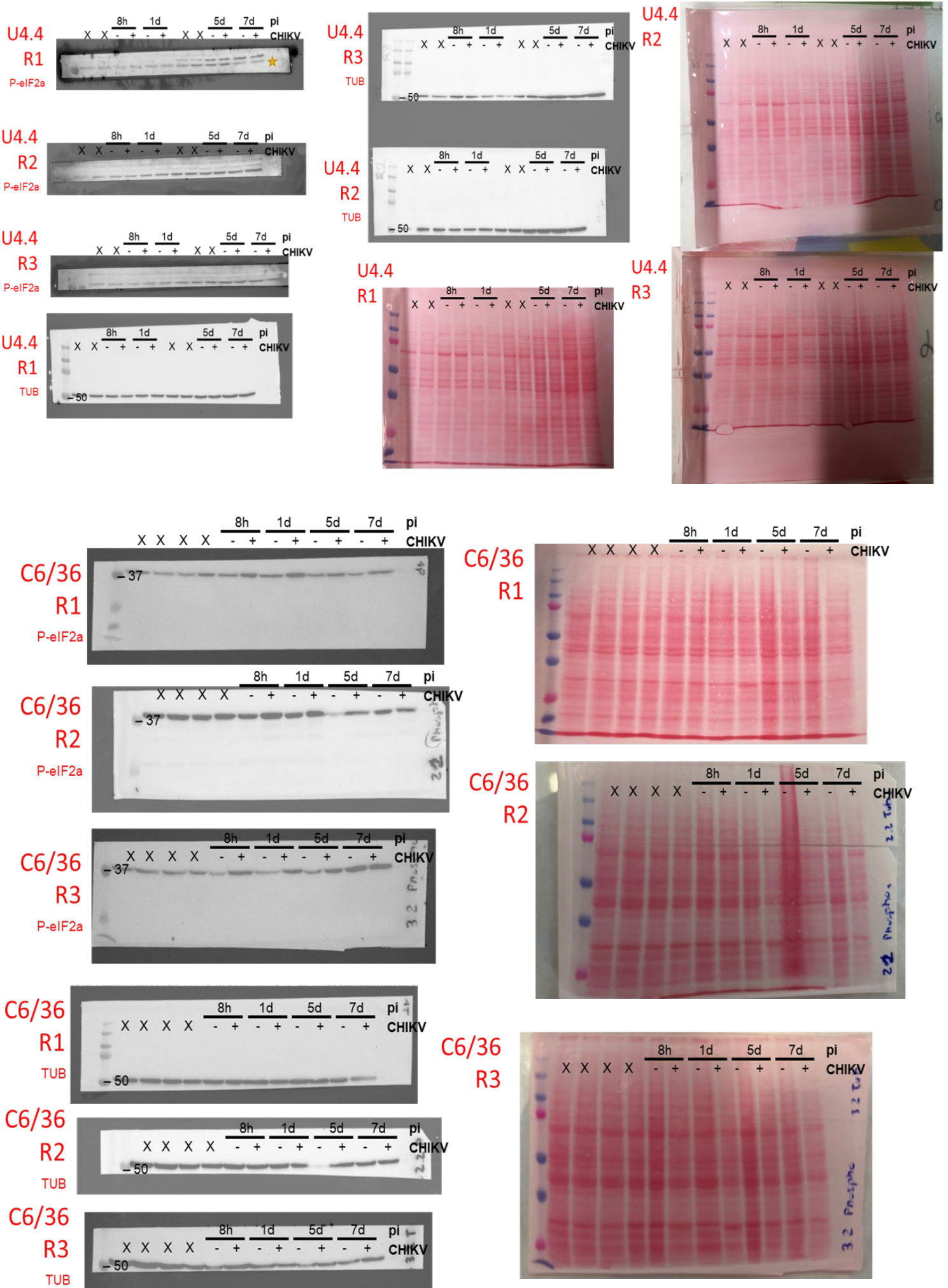

Fig 3A

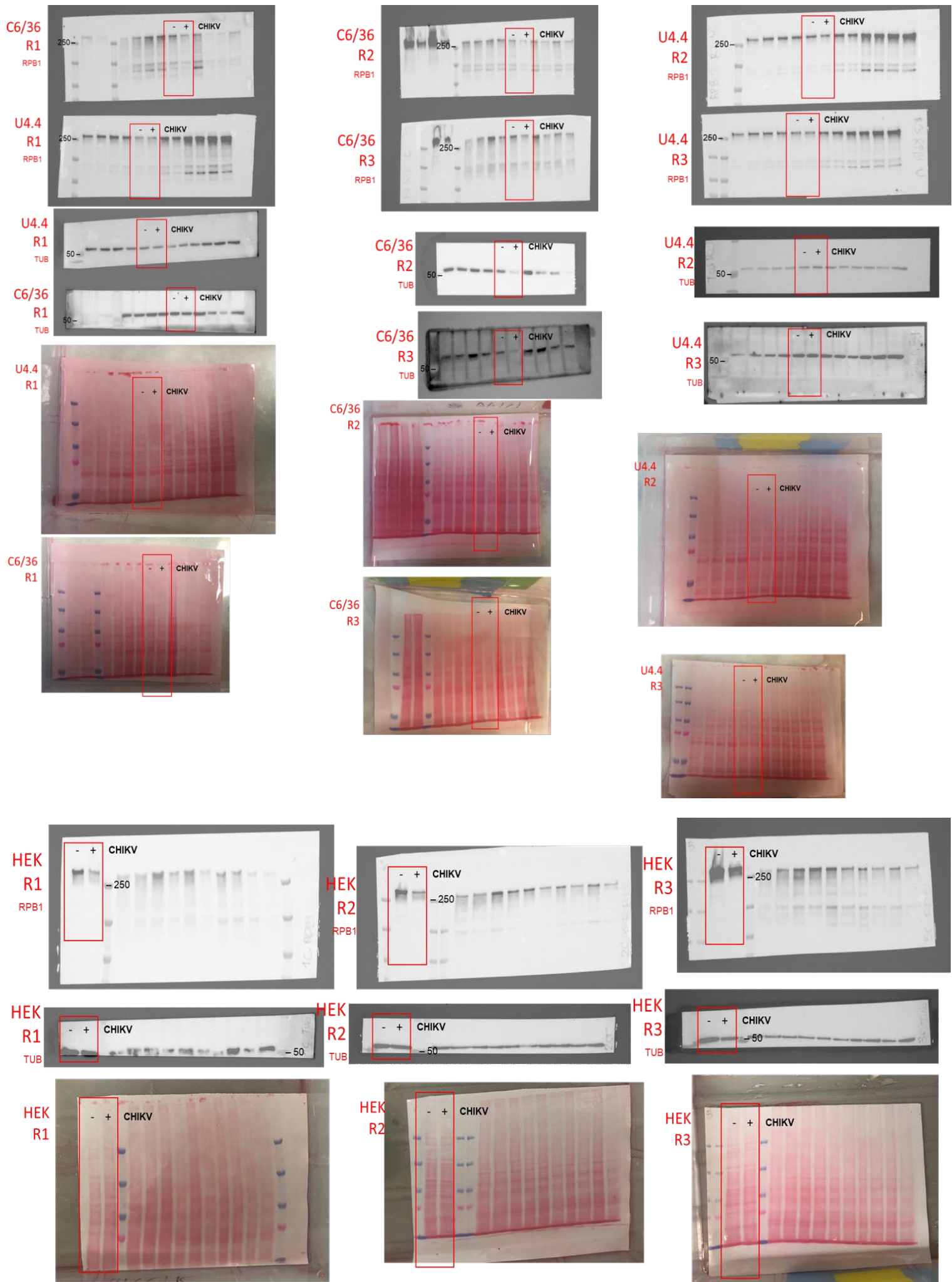

Fig 4D and Fig 4E

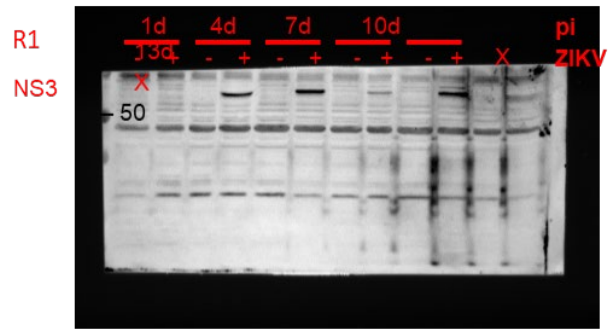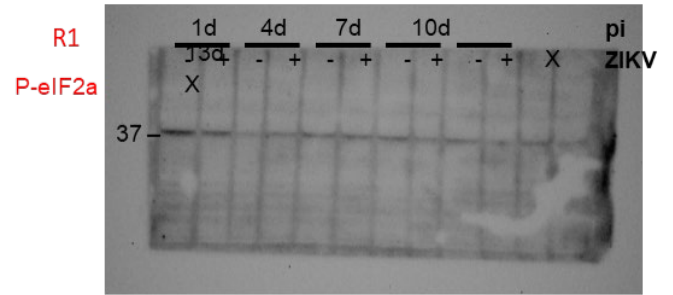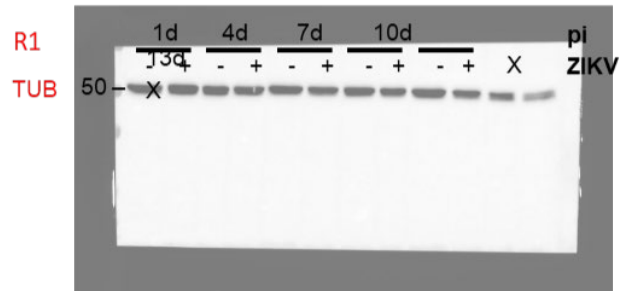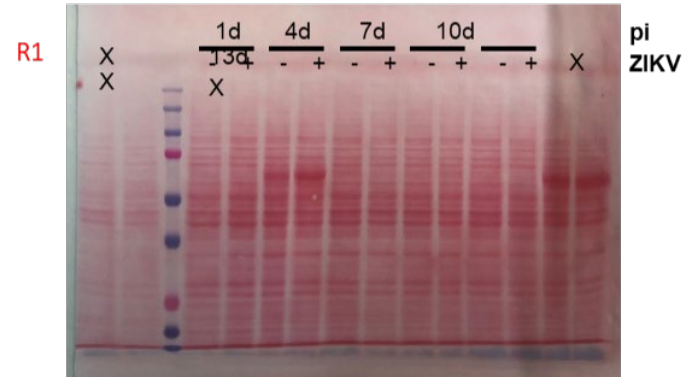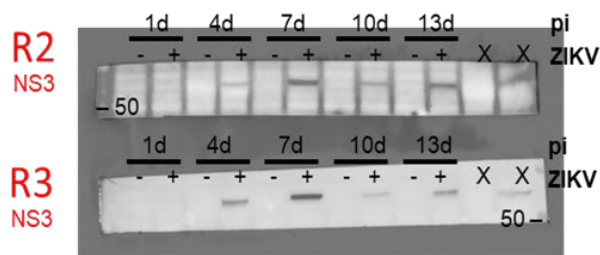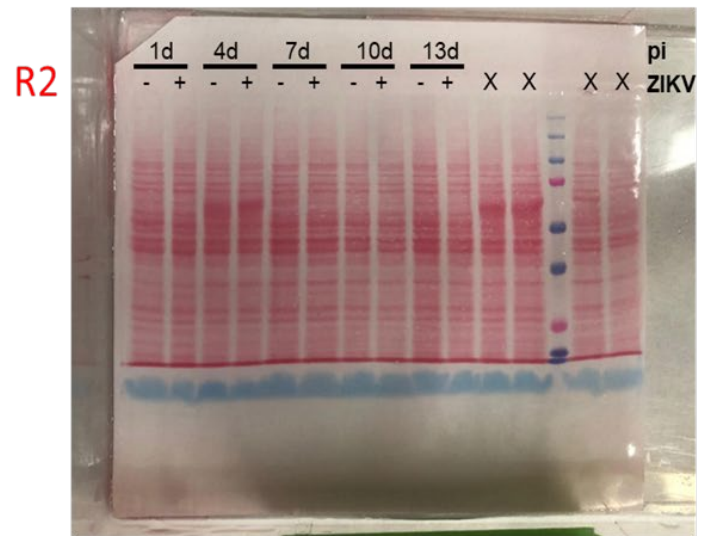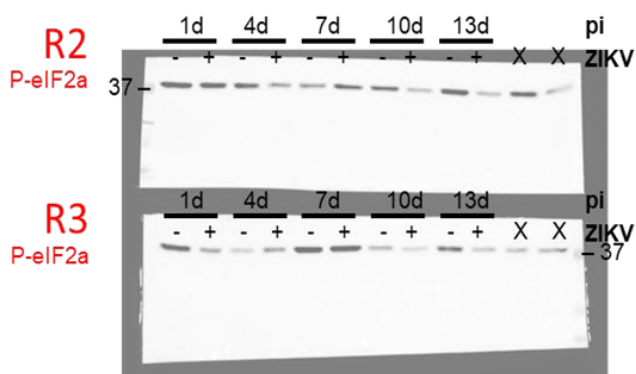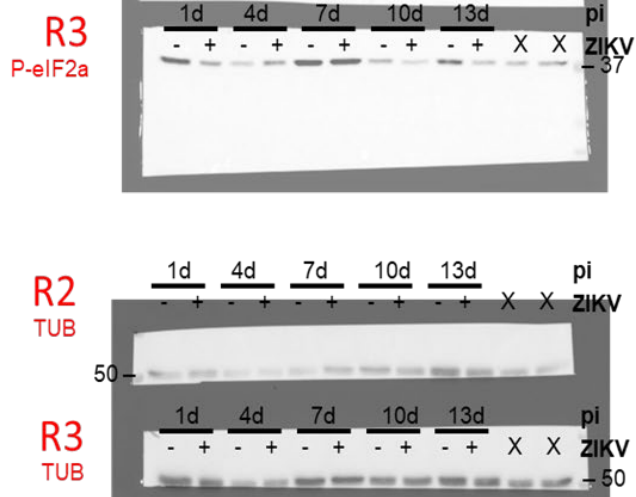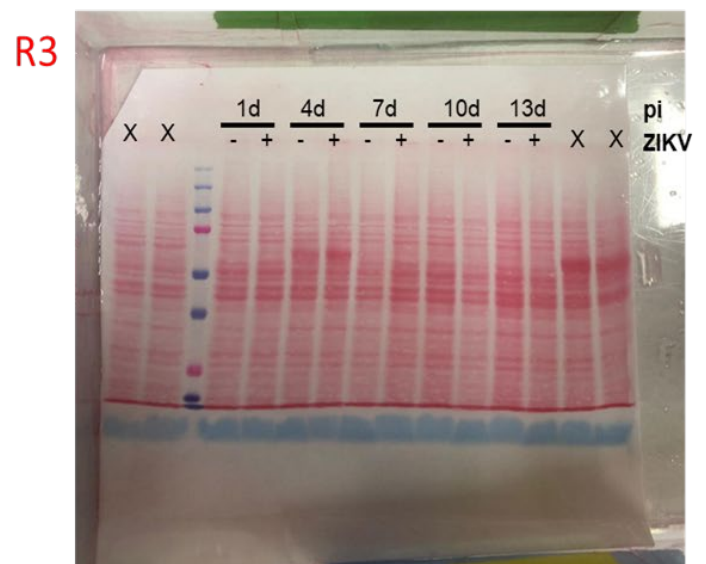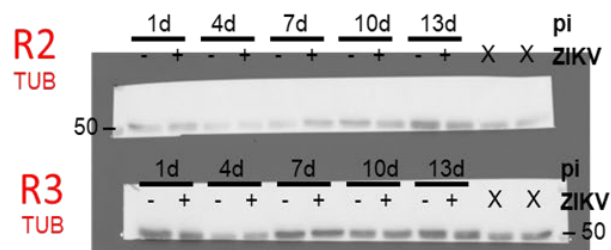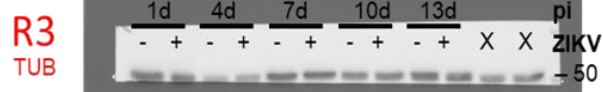

Supplement: S1 Raw Images — Original uncropped images supporting all blot and gel results. Separate pages show the images underlying Figs 1D–1E, 2E, 3A, and 4D–4E. (PDF) [file pbio.3003702.s004.pdf]
